# Effect of Hydroxyapatite Nanoparticles on the Ultrastructure, Developmental Competence, and Expression of *ZP3*, *MFN1*, and *NPM2* in Vitrified Bovine GV Oocytes

**DOI:** 10.3390/biology15060506

**Published:** 2026-03-21

**Authors:** Xiao-Xia Li, Shi-Yu Zhang, Jun Wang, Yi-Hang Wang, Jia-Hao Zhang, Shi-Han Zhao, Ping-Hua Cao, Yu-Mei Liu, Chen Zhou, Zhen Zhang, Qiao-Ting Shi, Waleid Mohamed EL-Sayed Shakweer, Ibrahim Mohamed EL-Sayed Shakweer, Zhi-Qian Xu

**Affiliations:** 1 College of Animal Science and Technology, Henan University of Science and Technology, Luoyang 471023, China; 2 Henan Provincial Key Laboratory for Grass-Feeding Animal, Henan University of Science and Technology, Luoyang 471023, China; 3 School of Information Engineering, Henan University of Science and Technology, Luoyang 471023, China; 4 Henan Seed Industry Development Center, Zhengzhou 450003, China; 5 Henan Provincial International Joint Laboratory for Dairy Health Farming, Zhengzhou 450003, China; 6 Institute of Animal Husbandry, Henan Academy of Agricultural Sciences, Zhengzhou 450002, China; 7 Agricultural and Biological Research Institute, National Research Centre, Dokki 12622, Egypt

**Keywords:** germinal vesicle oocytes, hydroxyapatite nanoparticles, cryoprotective agents, vitrification, ultrastructure, developmental competence, gene expression, bovine

## Abstract

To improve the vitrification efficiency of bovine germinal vesicle (GV) oocytes, the use of hydroxyapatite (HA) nanoparticles as a novel cryopreservation additive represents a promising approach. This study aimed to investigate the effects of HA nanoparticles and permeable cryoprotective agents (CPAs) on the ultrastructure, developmental competence, and gene expression of bovine GV oocytes following vitrification. Oocytes were vitrified in vitrification solutions containing HA nanoparticles of different sizes (20, 40, or 60 nm) and concentrations (0.01%, 0.05%, or 0.1%) to determine the optimal conditions based on survival rate, mitochondrial membrane potential (MMP) level, and developmental competence. Subsequently, the synergistic effects of HA nanoparticles and permeable CPAs (VS: 20% EG + 20% DMSO; VS1: 17.5% EG + 17.5% DMSO) were further evaluated. The optimal treatment (40 nm 0.05% HA nanoparticles) significantly increased MMP level, and improved developmental competence compared with the vitrified control group (*p* < 0.05). Among the vitrified groups, vitrified oocytes in the VS1-HA group (combining HA nanoparticles with reduced concentrations of permeable CPAs) exhibited the highest MMP level (1.89), maturation rate (50.39%), cleavage rate (27.07%), and blastocyst rate (10.53%) (*p* < 0.05). Ultrastructural analysis further revealed that the VS1-HA group maintained more intact zona pellucida structures and showed reduced mitochondrial swelling compared with the vitrified control group. Moreover, the expression levels of genes associated with zona pellucida formation (*ZP3*), mitochondrial fusion (*MFN1*), and chromatin remodeling (*NPM2*) were significantly upregulated in the VS1-HA group relative to the vitrified control group. Overall, these findings indicate that the combination of HA nanoparticles with lower concentrations of permeable CPAs enhances MMP level, alleviates vitrification-induced ultrastructural damage, and upregulates the expression of key developmental genes, thereby improving the developmental competence of vitrified bovine GV oocytes.

## Simple Summary

The cryopreservation efficiency of bovine germinal vesicle (GV) oocytes remains relatively low. Although hydroxyapatite (HA) nanoparticles have been investigated for use in cryopreservation, the effects of combining HA nanoparticles with reduced concentrations of permeable cryoprotective agents (CPAs) on bovine GV oocytes remain unclear. This study therefore examined the synergistic effects of HA nanoparticles and permeable CPAs on the ultrastructure, developmental competence, and gene expression of bovine GV oocytes following vitrification. The results demonstrated that the combination of HA nanoparticles with lower concentrations of permeable CPAs increased mitochondrial membrane potential (MMP) level, enhanced developmental competence, alleviated vitrification-induced ultrastructural damage, and upregulated the expression of the related genes, *ZP3*, *MFN1*, and *NPM2*, in vitrified bovine GV oocytes. These findings provide experimental evidence supporting the development of nanomaterial-based cryopreservation strategies for bovine oocytes and may contribute to improvements in reproductive technologies and germplasm conservation.

## 1. Introduction

Oocyte cryopreservation plays a crucial role in preserving genetic biodiversity, supporting assisted reproduction, and enabling the rapid propagation of livestock in mammals [1,2]. Germinal vesicle (GV) oocytes have attracted increasing attention because their chromosomes remain enclosed within the nuclear membrane, and the meiotic spindle has not yet formed, which may provide structural advantages during cryopreservation [3]. However, the cryopreservation efficiency of GV oocytes remains relatively low [4], highlighting the need for more effective strategies to improve this technique. Compared with conventional slow freezing, vitrification has become the preferred method for oocyte cryopreservation, due to its simplicity, rapid cooling rate, high efficiency, and markedly reduced risk of ice crystal formation [5].

Vitrification relies on the rapid solidification of vitrification solutions containing high concentrations of permeable cryoprotective agents (CPAs) when directly exposed to liquid nitrogen (LN), thereby preventing ice crystal formation [6]. The success of vitrification largely depends on both the cooling rate and the high concentration of permeable CPAs (i.e., high viscosity) [7]. However, high concentrations of permeable CPAs can cause inherent chemical toxicity and induce osmotic stress during both the vitrification and warming processes [8]. These adverse effects may lead to cellular damage as evidenced by decreased mitochondrial membrane potential (MMP) level [9], ultrastructural alterations [10], and reduced developmental competence [11]. In addition, vitrification can trigger molecular changes in oocytes, and the expression of key genes has been as an indicator of cryoinjury [12]. For instance, *ZP3* mediates sperm–egg recognition during fertilization, and reduced expression may impair fertilization capacity and early embryonic development [13]. *MFN1* regulates mitochondrial fusion, and its downregulation disrupts mitochondrial dynamics (*MFN1*) and cellular energy production [14,15]. Furthermore, the maternal-effect gene *NPM2* plays an essential role in chromatin remodeling during early embryogenesis, and reduced expression has been associated with decreased blastocyst formation and poor oocyte quality [16]. Collectively, these alterations may compromise oocyte quality and developmental potential.

To minimize vitrification-induced damage to oocytes, an effective strategy is to increase the viscosity of the vitrification solution without relying excessively on high concentrations of permeable CPAs. Recently, nanomaterials have been explored as additives in vitrification solution because of their favorable biological and physicochemical properties [17,18]. Their high thermal conductivity and ability to increase relative viscosity can facilitate vitrification, enhance solution stability during rewarming, and suppress devitrification [19]. However, many metal or carbon-based nanoparticles may accumulate in reproductive tissues, and exert toxic effects [20,21], which limits their application in the reproductive field. In contrast, hydroxyapatite (HA) nanoparticles exhibit excellent biocompatibility and high biosafety, making them promising candidates for use in germ cell vitrification [22].

HA nanoparticles exhibit good dispersion stability, moderate thermal conductivity, and high biocompatibility [23]. Previous studies have shown that HA nanoparticles can achieve stable dispersion in cryopreservation media, where surface-exposed Ca^2+^ and PO_4_^3−^ interact with permeable CPAs and water molecules, thereby increasing solution viscosity and inhibiting ice crystal formation [24]. In addition, the thermal conductivity of HA nanoparticles facilitates uniform heat transfer during ultra-rapid cooling [25]. Although the effectiveness of HA nanoparticles is influenced by their physicochemical characteristics, such as particle size, surface charge, and hydrophobicity, their use as a cryoprotective additive may offer protective effects for oocytes during vitrification and warming [26]. HA nanoparticles with appropriate particle sizes and concentrations have been reported to improve the survival rate and enhance the developmental competence of vitrified GV oocytes in ovine and porcine [22,27]. Therefore, incorporating HA nanoparticles with suitable sizes and concentrations of vitrification solutions may theoretically allow for a reduction in the concentration of permeable CPAs during vitrification, thereby improving oocyte quality and subsequent developmental competence. However, previous studies have mainly focused on the effects of HA nanoparticle supplementation alone during oocyte vitrification [22,27,28], and the potential synergistic effects of combining HA nanoparticles with a lower concentration of permeable CPAs on the cryopreservation of bovine GV oocytes remain unclear.

Therefore, this study investigated the synergistic effects of HA nanoparticles and permeable CPAs on survival rate, MMP level, developmental competence, ultrastructure, and the expression of key genes (*ZP3, MFN1*, and *NPM2*) in bovine GV oocytes following vitrification–warming. The aim was to explore the potential mechanisms underlying the protective role of this combined approach against vitrification-induced damage.

## 2. Materials and Methods

### 2.1. Reagents and Supplies

Tissue culture medium 199 (TCM-199) was obtained from Gibco BRL (Grand Island, NY, USA), fetal bovine serum (FBS) was purchased from Zhejiang Tianhang Biotechnology Co., Ltd. (Hangzhou, China), and HA nanoparticles were obtained from Nanjing Emperor Nano Material Co., Ltd. (Nanjing, China). BO-HEPES-IVM, BO-IVF, and BO-IVC media were purchased from IVF Bioscience Co. (St. Falmouth, Cornwall, UK). Unless otherwise specified, all other reagents were obtained from Sigma-Aldrich (St. Louis, MO, USA).

### 2.2. Experimental Design and Treatment Groups

Five independent experiments were conducted sequentially to systematically evaluate the effects of HA nanoparticles on vitrified bovine GV oocytes. A summary of all treatment groups is presented in Table 1.

#### 2.2.1. Experiment 1: Assessment of Intracellular Localization, Dispersion Stability, and Toxicity of HA Nanoparticles

Transmission electron microscopy (TEM) was used to determine whether HA nanoparticles could enter the oocytes. Subsequently, 0.1% HA nanoparticles of different particle sizes (20, 40, and 60 nm) were separately added to vitrification solution and subjected to ultrasonic treatment for different durations (0, 1, 2, 3, 4, 5, and 6 min). The absorbance of each group was then measured, and the experiment was repeated three times. GV oocytes were randomly assigned to five groups: Fresh, VS, VH20, VH40, and VH60. Except for the Fresh group, oocytes in the other groups were vitrified using their corresponding vitrification solutions. After warming, a total of 256, 260, and 766 GV oocytes were used to evaluate the survival rate, MMP level, and maturation rate, respectively, in order to identify the optimal nanoparticle size for subsequent experiments. Each experiment was independently repeated at least three times.

#### 2.2.2. Experiment 2: Effects of Different Concentrations of HA Nanoparticles on Developmental Competence in Vitrified Bovine GV Oocytes

The developmental competence of vitrified bovine GV oocytes treated with different concentrations of HA nanoparticles was evaluated based on survival rate, MMP level, maturation rate, cleavage rate, and blastocyst rate. GV oocytes were randomly assigned to five groups: Fresh, VS, VH-0.01%, VH-0.05%, and VH-0.1%. Except for the Fresh group, oocytes in the remaining groups were vitrified using their respective vitrification solutions. After warming, a total of 286, 255, and 684 GV oocytes were used to assess the survival rate, MMP level, and developmental competence, respectively. Each experiment was independently repeated at least three times. Based on these results, the optimal concentration of HA nanoparticles was selected and applied in the subsequent experiments.

#### 2.2.3. Experiment 3: Evaluation of the Synergistic Effect Between HA Nanoparticles and Different Concentrations of Permeable CPAs on Developmental Competence in Vitrified Bovine GV Oocytes

The developmental competence of vitrified bovine GV oocytes treated with a combination of HA nanoparticles and different concentrations of permeable CPAs was evaluated based on survival rate, MMP level, maturation rate, cleavage rate, and blastocyst rate. GV oocytes were randomly assigned to five groups: Fresh, VS, VS1, VS1-HA, and VS-HA. Except for the Fresh group, oocytes in the remaining groups were vitrified using their respective vitrification solutions. After warming, a total of 294, 261, and 673 oocytes were used to assess the survival rate, MMP level, and developmental competence, respectively. Each experiment was independently repeated at least three times.

#### 2.2.4. Experiment 4: Effect of the Synergy Between HA Nanoparticles and Different Concentrations of Permeable CPAs on Ultrastructure in Vitrified Bovine GV Oocytes

GV oocytes were collected and randomly assigned to four groups: Fresh, VS, VS1-HA, and VS-HA. Following vitrification and warming, GV oocytes from each group were incubated for 24 h to allow for maturation. Subsequently, each group, consisting of at least 50 oocytes, was processed into ultrathin sections for ultrastructural examination using TEM.

#### 2.2.5. Experiment 5: Effect of the Synergy Between HA Nanoparticles and Different Concentrations Permeable CPAs on Related Gene Expression of Vitrified Bovine GV Oocytes

To evaluate the synergistic effects of HA nanoparticles and different concentrations of permeable CPAs on the expression of *ZP3*, *MFN1*, and *NPM2*, a total of 600 GV oocytes were randomly assigned to four groups, as in Experiment 4, and subjected to vitrification and warming. The oocytes were then cultured for 24 h to allow for maturation. Hyaluronidase was subsequently used to completely remove the surrounding granulosa cells, enabling assessment of the selected genes. Each experimental group was independently repeated three times.

### 2.3. Intracellular Localization and Dispersion Stability of HA Nanoparticles

TEM was employed to investigate the localization of HA nanoparticles within oocytes, as detailed in Section 2.7. A 0.1% (*w*/*v*) suspension of HA nanoparticles with particle sizes of 20, 40, and 60 nm was added to the vitrification solution (VS; formulation provided in the footnote of Table 1) and pre-mixed using magnetic stirring at 800 rpm for 5 min. Samples (200 µL, *n* = 3) were then subjected to sonication in an ice bath using an ultrasonic cell crusher (UH-500A, Tianjin Automatic Science Instruments Co., Ltd., Tianjin, China). Ultrasonic pulse treatment was performed for varying durations (0, 1, 2, 3, 4, 5, and 6 min) at 500 W power and 80 Hz frequency. Following sonication, the samples were immediately transferred to a 96-well plate, and absorbance at 360 nm was measured using a microplate reader (Tecan Infinite 200 Pro, Tecan Group Ltd., Männedorf, Switzerland). These absorbance values were used to determine the optimal ultrasonic treatment duration and to assess the dispersion stability of HA nanoparticles of different sizes (20, 40, and 60 nm).

### 2.4. Oocytes Collection

Bovine ovaries were obtained from local commercial slaughterhouses and transported to the laboratory within 4 h in sterile physiological saline solution containing 100 IU/mL penicillin and 100 µg/mL of streptomycin sulfate at 35 °C. Bovine GV oocytes were then aspirated from follicles measuring 2–8 mm in diameter, using a 10 mL syringe fitted with a 12-gauge needle at room temperature. Oocytes surrounded by more than three layers of compact cumulus cells and exhibiting uniform cytoplasm were selected and maintained in M-199 supplemented with 3% (*v*/*v*) FBS for subsequent experiments.

### 2.5. Vitrification and Warming of Oocytes

#### 2.5.1. Preparation of Vitrification and Warming Solutions

A series of specialized media were used during the vitrification and warming procedures. The holding medium (HM) consisted of TCM-199 supplemented with 20% (*v*/*v*) FBS. The pre-vitrification solution was prepared by adding 10% (*v*/*v*) ethylene glycol (EG) and 10% (*v*/*v*) dimethyl sulfoxide (DMSO) to HM. For vitrification, two different types of solutions were used: (1) vitrification solution (VS): 60% (*v*/*v*) HM, 20% (*v*/*v*) EG, 20% (*v*/*v*) DMSO, and 0.5 M sucrose; (2) vitrification solution I (VS1): 70% (*v*/*v*) HM with 17.5% (*v*/*v*) EG, 17.5% (*v*/*v*) DMSO and 0.5 M sucrose. Two warming solutions were also used: (1) warming solution I (W1): HM supplemented with 0.5 M sucrose; (2) warming solution II (W2): HM supplemented with 0.25 M sucrose. All solutions were pre-warmed to 38 °C prior to use.

#### 2.5.2. Vitrification and Warming

GV oocytes were vitrified using the open pulled straw (OPS) method [29]. All oocyte manipulations were performed on a hot plate (HP-4530N, AS ONE, Tokyo, Japan) maintained at 38 °C. Briefly, 7-10 GV oocytes were first incubated in HM for 5 min, then transferred to pre-vitrification solution for 1 min, and subsequently placed into a 10 μL microdroplet of vitrification solution with or without HA nanoparticles (specific formulations for each treatment group are provided in Table 1). Following rapid penetration with a 1 μL microdroplet of the same solution, the GV oocytes were immediately loaded into an OPS (IMV Technologies, L’Aigle, France) and immersed in LN within 30 s. This OPS was then placed into a frozen bag, labeled, and stored in an LN tank (YDS-20, Xinxiang Xinya Low Temperature Co., Ltd, Xinxiang, China).

Vitrified oocytes were warmed following a standardized protocol [12]. The OPS was quickly retrieved from the LN tank, and its narrow end was immediately immersed in a 50 μL W1 microdroplet. While the wider end was manually occluded, GV oocytes were expelled into the solution through thermally induced expansion. The oocytes were then sequentially transferred to W1, W2, and HM, with 5 min of incubation at each step. Finally, the warmed oocytes were prepared for subsequent experimental analyses.

### 2.6. Trypan Blue Staining

The survival rate of oocytes following vitrification and warming was assessed using trypan blue staining (Lanjieke Biotechnology Co., Ltd., Beijing, China). After warming, GV oocytes from the vitrified groups were treated with hyaluronidase (100 IU/mL) for 2 min to remove cumulus cells and subsequently washed three times in phosphate-buffered saline (PBS) containing 0.5% bovine serum albumin. The denuded oocytes were then incubated with 0.4% trypan blue for 5 min in the dark and washed with PBS. Oocytes with compromised plasma membranes indicating cell death were stained blue due to dye penetration, whereas viable oocytes with intact membranes remained unstained. Survival rate was calculated as the percentage of unstained oocytes relative to the total number assessed. Each experimental group was independently replicated three times, with a minimum of 15 oocytes per group.

### 2.7. JC-1 Dual Staining

The MMP level of bovine GV oocytes was assessed using a JC-1 assay kit (Beyotime Biotechnology, Shanghai, China). After treatment with hyaluronidase (100 IU/mL) for 2 min, the surrounding cumulus cells were removed from the oocytes. Fully denuded oocytes from both Fresh and vitrified–warmed groups were washed in PBS and incubated with 0.5 µM JC-1 for 30 min at 38.5 °C in the dark. Following incubation, oocytes were washed with JC-1 staining buffer, and gently transferred onto glass slides. To minimize mechanical stress, the slides were pre-coated with Vaseline circles to confine the sample, and coverslips were applied carefully without pressure. JC-1 fluorescence (red/green) was observed at 100× magnification using an inverted fluorescence microscope (Axio Observer, A1, Carl Zeiss AG, Oberkochen, Germany). The red-to-green fluorescence ratio was quantified using ImageJ software (version 1.54p, NIH, Bethesda, MD, USA) to evaluate MMP level. Each experimental group included at least 15 oocytes and was independently repeated three times.

### 2.8. In Vitro Maturation (IVM)

Following warming, GV oocytes from both Fresh and vitrified–warmed groups were selected and washed three times in maturation medium (BO-HEPES-IVM). Subsequently, 12–15 GV oocytes were cultured in a 100 μL microdroplet of BO-HEPES-IVM under mineral oil for 24 h at 38.5 °C in humidified air atmosphere containing 5% CO_2_. After IVM, the proportion of oocytes exhibiting the first polar body was recorded to determine the maturation rate.

### 2.9. In Vitro Fertilization (IVF) and In Vitro Culture (IVC)

After IVM, matured oocytes from the Fresh and vitrified–warmed groups were washed three times in BO-IVF medium and randomly allocated to 90 µL microdroplets of the same medium covered with mineral oil, with 12–15 oocytes per droplet. Meanwhile, frozen–thawed semen from an Angus bull (No. 41413622) was added to BO-IVF medium and pre-incubated for 45 min at 38.5 °C in a humidified atmosphere containing 5% CO_2_ [30]. A 10 μL sperm suspension (1 × 10^7^ sperm/mL) was then added to microdroplets of BO-IVF and co-incubated with the oocytes for 6–8 h at 38.5 °C in 5% CO_2_ humidified air.

Following IVF, presumptive zygotes were washed and cultured in BO-IVC medium (12–15 zygotes per 100 μL microdroplet) under a humidified atmosphere with 5% CO_2_ at 38.5 °C. The cleavage rate was recorded on Day 2, and the blastocyst rate was evaluated between Days 7 and 10 of culture, with Day 0 defined as the day of IVF. Note: Only early and expanded blastocysts were included in the calculation of the blastocyst rate for statistical analysis.

### 2.10. TEM

GV oocytes from the Fresh, VS, VS1-HA, and VS-HA groups were cultured for 24 h following vitrification and warming. After maturation, the cumulus cells surrounding the oocytes were gently remove using a 200 μL pipette. The denuded oocytes were then washed three times in PBS, fixed in 4% glutaraldehyde at 4 °C and post-fixed in 1% osmium tetroxide for 2 h. Samples were subsequently dehydrated through a graded ethanol series and embedded in Epon-812 resin. Ultrathin sections (60–90 nm) were prepared using a microtome (UC7rt, Leica, Wetzlar, Germany), mounted on copper grids, and stained with uranyl acetate and lead citrate. Finally, the sections were examined using TEM (JEM-1400FLASH, JEOL, Tokyo, Japan).

### 2.11. RNA Extraction, cDNA Synthesis, and Real-Time Quantitative PCR (RT-qPCR)

Following vitrification and warming, GV oocytes from the Fresh, VS, VS1-HA, and VS-HA groups were cultured for 24 h to allow maturation. Approximately 50 matured oocytes per group were treated with hyaluronidase (100 IU/mL) to completely remove the surrounding cumulus cells. Total RNA was then extracted using the TRIGene Plus kit (GenStar, Beijing, China) according to the manufacturer’s instructions, involving sequential processing with TRIGene reagent, isopropanol, and 75% ethanol. The purified RNA was finally dissolved in 11 μL nuclease-free water. Complementary DNA (cDNA) synthesis was performed using the StarScript Pro All-in-one RT Mix with the gDNA Remover kit (GenStar, Beijing, China) in a 20 μL reaction system comprising 1 μL of 5× StarScript Pro All-in-one RT Mix, 4 μL of 5× StarScript Pro All-in-one RT Buffer, 10 μL of total RNA, and 5 μL of nuclease-free water. Reverse transcription was carried out using the following program: 37 °C for 2 min, 50 °C for 10 min, and 85 °C for 2 min, followed by immediate cooling on ice.

RT-qPCR was performed on a CFX Connect™ Real-Time PCR Detection System (Bio-Rad, Hercules, CA, USA) using TB Green^®^ Premix Ex Taq™ II (Takara, Dalian, China). Each amplification reaction was carried out in a 12.5 μL reaction mixture containing 6.25 μL of TB Green Premix Ex Taq II, 1 μL of cDNA, 1 μL of primers, and 4.25 μL of ultrapure water. A negative control was included by replacing the cDNA template with an equal volume of ultrapure water.

All primers were synthesized by Sangon Biotech Co., Ltd. (Zhengzhou, China) and sequences with corresponding information are listed in Table 2. PCR amplification followed a standard two-step cycling protocol: initial denaturation at 95 °C for 30 s, followed by 40 cycles of denaturation at 95 °C for 5 s and annealing/extension at 60 °C for 30 s. A melt curve analysis was performed after amplification to verify the specificity of the PCR products. The expression levels of the target gene were normalized to the reference gene *β-actin*, which exhibited stable Cq values across all oocyte samples.

The relative mRNA expression levels of the target gene were calculated using the 2^−∆∆Ct^ method and expressed as fold changes relative to the Fresh group. The magnitude of gene expression change (X) was determined using the formula X = 2^−∆∆Ct^, where ∆∆Ct = (Ct_target gene_ − Ct*_β-actin_*) sample − (Ct_target gene_ − Ct*_β-actin_*) control [27].

### 2.12. Statistical Analyses

All statistical analyses were performed using IBM SPSS Statistics (version 25.0, IBM Corp., Armonk, NY, USA). One-way analysis of variance (ANOVA) was used to evaluate differences among groups for oocyte survival rate, MMP level, developmental competence, and gene expression. For the assessment of HA nanoparticle dispersion stability, a two-way ANOVA was conducted to evaluate the effects of HA nanoparticle particle size and ultrasonic treatment duration. Significant differences among groups were further analyzed using Duncan’s multiple range test for multiple comparisons. Unless otherwise stated, data are presented as the mean ± standard deviation (SD), and statistical significance was set at *p* < 0.05.

## 3. Results

### 3.1. Experiment 1: Assessment of Intracellular Localization, Dispersion Stability, and Toxicity of HA Nanoparticles

**Figure 1:**
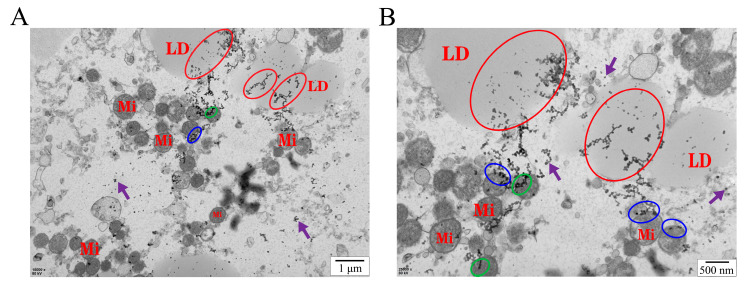
Transmission electron microscopy (TEM) images showing the intracellular localization of HA nanoparticles in vitrified bovine GV oocytes after IVM. Scale bars: 1 µm (**A**), 500 nm (**B**). Labels: mitochondrion (Mi), lipid droplet (LD). Markers: red circle: HA nanoparticles on lipid droplets; blue circle: HA nanoparticles on the outer membrane of mitochondria; green circle: HA nanoparticles on mitochondria; purple arrow: HA nanoparticles.

**Figure 2:**
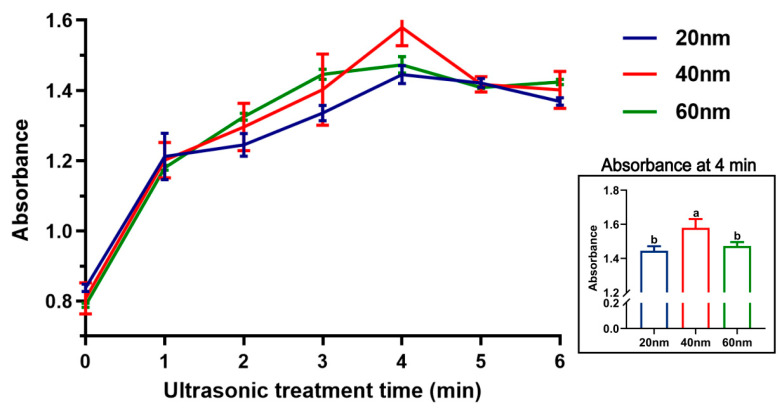
Absorbance of HA nanoparticles (20, 40, and 60 nm, 0.1% *w*/*v*) measured at different ultrasonic treatment times. The inset shows absorbance values at 4 min. Different lowercase letters (a, b) indicate statistically significant differences (*p* < 0.05).

**Figure 3:**
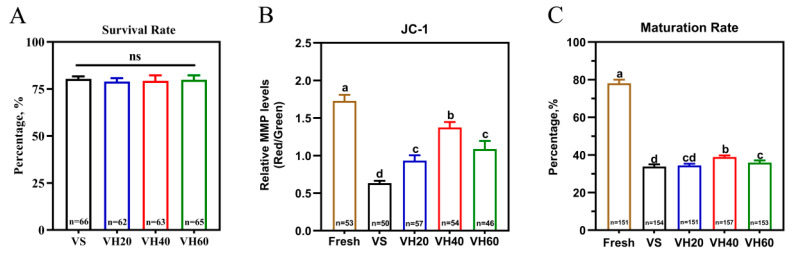
Effects of HA nanoparticle size on survival rate, mitochondrial membrane potential (MMP) level and maturation rate of vitrified–warmed bovine GV oocytes. (**A**) Survival rate of vitrified–warmed GV oocytes. (**B**) Quantitative analysis of MMP (JC-1 red/green fluorescence ratio). (**C**) Maturation rate of vitrified–warmed GV oocytes after IVM. Data presentation: mean ± SD; ns: not significant (*p* > 0.05); different lowercase letters (a, b, c, d) indicate significant differences (*p* < 0.05). Experimental groups: Fresh, non-vitrified control; VS, vitrified control without HA nanoparticles; VH20, vitrified with 20 nm HA nanoparticles; VH40, vitrified with 40 nm HA nanoparticles; VH60, vitrified with 60 nm HA nanoparticles.

TEM analysis revealed that HA nanoparticles were internalized by the oocytes, appearing both as aggregated clusters and scattered individual particles (Figure 1). The vast majority of HA nanoparticles were localized on the surface of lipid droplets; some were associated with mitochondria, and a small fraction was sparsely distributed within the cytoplasm. The absorbance of vitrification solutions subjected to different ultrasonic treatment times is shown in Figure 2. After 4 min of ultrasonic treatment, the absorbance of 40 nm HA nanoparticles (1.58) was significantly higher (*p* < 0.05) than that of 20 nm (1.44) and 60 nm (1.47) nanoparticles, indicating that dispersion stability was optimal at a particle size of 40 nm with 4 min of ultrasonic treatment. The post-warming survival rate of bovine GV oocytes did not differ significantly between groups treated with HA nanoparticles and the VS group (*p* > 0.05) (Figure 3A). However, the MMP level was significantly higher in the VH40 group (1.37) compared with the VS (0.63), VH20 (0.93), and VH60 (1.09) groups (*p* < 0.05) (Figure 3B). Similarly, following IVM, the maturation rate in the VH40 group (38.85%) was significantly higher than in the VS (33.77%), VH20 (34.44%), and VH60 (35.95%) groups (*p* < 0.05) (Figure 3C).

These findings indicate that HA nanoparticle treatment did not negatively affect the survival rate, MMP level, or maturation rate of vitrified GV oocytes compared with the VS group, demonstrating the biosafety of HA nanoparticles under the experimental conditions. Based on these results, 40 nm was selected as the optimal particle size for subsequent experiments.

### 3.2. Experiment 2: Effects of Different Concentrations of HA Nanoparticles on Developmental Competence in Vitrified Bovine GV Oocytes

**Figure 4:**
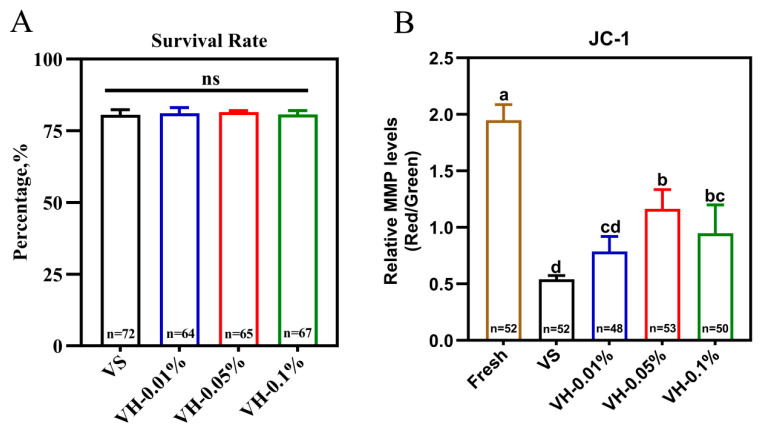
Effects of different concentrations of HA nanoparticles on survival rate and MMP level of vitrified bovine GV oocytes (**A**) Survival rate of vitrified–warmed GV oocytes. (**B**) Quantitative analysis of MMP (JC-1 red/green fluorescence ratio). Data presentation: mean ± SD; ns: no significant (*p* > 0.05); different lowercase letters (a, b, c, d) indicate significant differences (*p* < 0.05). Experimental groups: Fresh, non-vitrified control; VS, vitrified control without HA nanoparticles; VH-0.01%, vitrified with 40 nm 0.01% HA nanoparticles; VH-0.05%, vitrified with 40 nm 0.05% HA nanoparticles; VH-0.1%, vitrified with 40 nm 0.1% HA nanoparticles.

In this experiment, post-warming survival rates did not differ significantly among the vitrified groups (*p* > 0.05) (Figure 4A). However, the MMP level in the VH-0.05% group (1.16) was significantly higher than in the VH-0.01% (0.79) and VH-0.1% (0.95) groups (*p* < 0.05) (Figure 4B). The subsequent development of oocytes in the Fresh, VS, VH-0.01%, VH-0.05%, and VH-0.1% groups is summarized in Table 3. The VH-0.05% group exhibited a significantly higher maturation rate, cleavage rate, and blastocyst rate compared with the VS group, as well as the VH-0.01% and VH-0.1% groups (*p* < 0.05, Table 3). Based on these results, 40 nm HA nanoparticles at 0.05% concentration were elected for inclusion in the vitrification solutions for subsequent experiments.

### 3.3. Experiment 3: Evaluation of the Synergistic Effect Between HA Nanoparticles and Different Concentrations Permeable CPAs on Developmental Competence in Vitrified Bovine GV Oocytes

**Figure 5:**
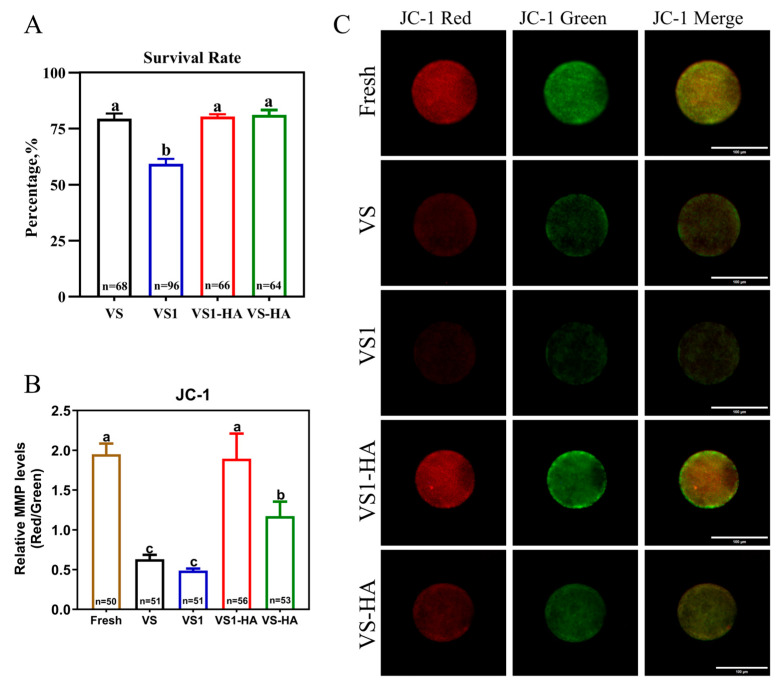
Effects of HA nanoparticles combined with different concentrations of permeable CPAs on survival rate and MMP level of vitrified bovine GV oocytes. (**A**) Survival rate of vitrified–warmed GV oocytes. (**B**) Quantitative analysis of MMP (JC-1 red/green fluorescence ratio). (**C**) Fluorescence images of mitochondrial distribution. Merged channels showing mitochondrial colocalization (green: JC-1 monomer; red: JC-1 aggregates). Scale bar = 100 µm. Data presentation: Mean ± SD; different lowercase letters (a, b, c) indicated significant differences (*p* < 0.05). Experimental groups: Fresh, non-vitrified control; VS, vitrified control without HA nanoparticles (CPAs: 20% EG + 20% DMSO); VS1, vitrified with 17.5% EG and 17.5% DMSO, without HA nanoparticles; VS1-HA, vitrified with 17.5% EG, 17.5% DMSO, and 40 nm 0.05% HA nanoparticles; VS-HA, vitrified with 20% EG, 20% DMSO, and 40 nm 0.05% HA nanoparticles.

The MMP level and survival rate of oocytes in the Fresh, VS, VS1, VS1-HA, and VS-HA groups are presented in Figure 5, with their subsequent development summarized in Table 4. The MMP level in the VS1-HA group (1.89) was significantly higher than in the VS (0.63) and VS-HA (1.17) groups (*p* < 0.05), and was comparable to the Fresh (1.95) group (*p* > 0.05%). Similarly, the VS1-HA group exhibited the highest maturation rate, cleavage rate, and blastocyst rate among the vitrified groups (*p* < 0.05). In contrast, oocytes in the VS1 group showed markedly reduced developmental competence despite similar mitochondrial activity, with survival rate (58.13% vs. 79.42%), maturation rate (25.81% vs. 38.55%), cleavage rate (7.10% vs. 10.94%) and blastocyst rate (1.94% vs. 3.13%) all significantly lower than those in the VS group. These results indicate that GV oocytes in the VS1 group suffered impaired developmental capacity. Due to the significantly lower survival rate and developmental competence in the VS1 group compared with the vitrified control, coupled with the limited number of oocytes available per collection day, this treatment was excluded from subsequent experiments.

Collectively, these results indicate that the combination of HA nanoparticles with lower concentrations of permeable CPAs significantly enhances the developmental competence of vitrified bovine GV oocytes.

### 3.4. Experiment 4: Effect of the Synergy Between HA Nanoparticles and Different Concentrations of Permeable CPAs on Ultrastructure in Vitrified Bovine GV Oocytes

**Figure 6:**
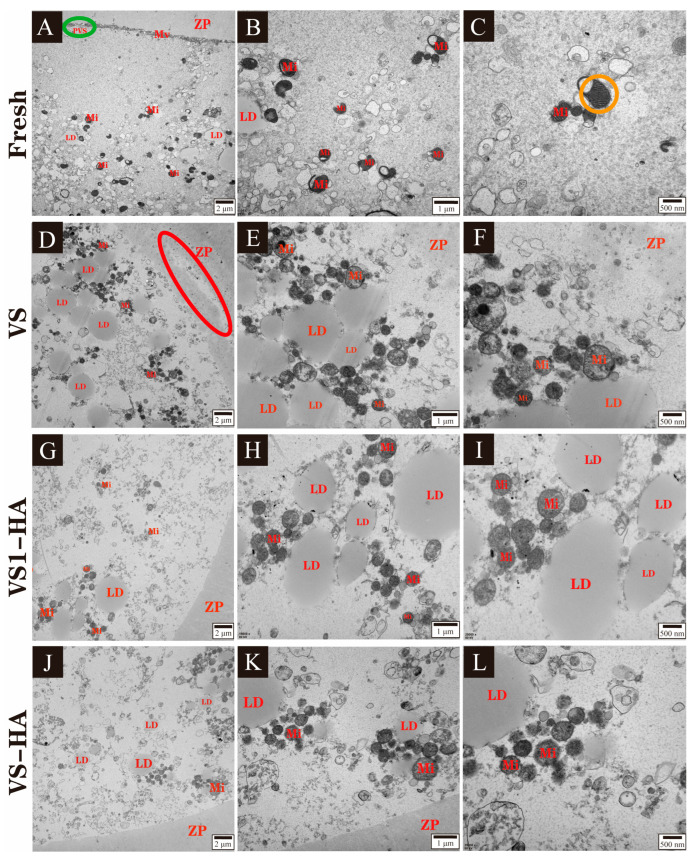
Transmission electron micrographs (TEM) of vitrified–warmed bovine GV oocytes after IVM from different experimental groups. Each row represents one treatment group: Fresh: non-vitrified control; VS: vitrified control without HA nanoparticles (CPAs: 20% EG + 20% DMSO); VS1-HA: vitrified with 17.5% EG, 17.5% DMSO, and 40 nm 0.05% HA nanoparticles; VS-HA: vitrified with 20% EG, 20% DMSO, and 40 nm 0.05% HA nanoparticles. Ultrastructural images are shown from left to right in increasing magnification. Oocytes of the Fresh group were observed with scale bars of 2 µm (**A**), 1 µm (**B**), and 500 nm (**C**); oocytes of the VS group were observed with scale bars of 2 µm (**D**), 1 µm (**E**), and 500 nm (**F**); oocytes of the VS1-HA group were observed with scale bars of 2 µm (**G**), 1 µm (**H**), and 500 nm (**I**); oocytes of the VS-HA group were observed with scale bars of 2 µm (**J**), 1 µm (**K**), and 500 nm (**L**). Identified structures include mitochondrion (Mi), lipid droplets (LD), zona pellucida (ZP), microvilli (Mv), and the perivitelline space (PVS). Markers: green circle: PVS; orange circle: mitochondria with intact transverse ridges; red circle: the partially dissolved ZP.

TEM revealed distinct ultrastructural features in matured oocytes across the different treatment groups (Figure 6). Oocytes in the Fresh group displayed normal ultrastructure: the zona pellucida was uniform with smooth edges, and the perivitelline space (green circle) and abundant microvilli were clearly visible (Figure 6A). Mitochondria exhibited intact cristae with clear transverse ridges (orange circles, Figure 6C).

Following vitrification, oocytes exhibited ultrastructural damage of varying severity. In all vitrified groups, the perivitelline space and microvilli disappeared. In the VS group, oocytes showed partial dissolution and irregular margins of the zona pellucida (red circle), and numerous lipid droplets were present in the cytoplasm (Figure 6D). Mitochondria were extensively swollen, with severe disruption of cristae (Figure 6F). By contrast, oocytes in the VS1-HA group displayed markedly reduced vitrification-induced damage. The zona pellucida remained uniform with smooth edges, and only a small number of lipid droplets were observed in the cytoplasm (Figure 6G). Mitochondria exhibited only mild swelling (Figure 6H). In the VS-HA group, the zona pellucida was generally uniform but had slightly irregular margins, and a few lipid droplets were visible within the cytoplasm (Figure 6J). Mitochondria showed mild swelling with minor fragmentation and partial dissolution of cristae (Figure 6K,L). These observations indicate that combining HA nanoparticles with lower concentrations of permeable CPAs mitigates vitrification-induced ultrastructural damage in bovine GV oocytes.

Vitrification induced pronounced ultrastructural damage in bovine oocytes, including partial dissolution of the zona pellucida, loss of microvilli, lipid accumulation, and mitochondrial swelling. In contrast, oocytes treated with HA nanoparticles, particularly those in the VS1-HA group, exhibited relatively well-preserved ultrastructure with minimal organelle abnormalities. These findings indicate that HA nanoparticles effectively synergize with low-concentration permeable CPAs to mitigate vitrification-induced damage in bovine oocytes.

### 3.5. Experiment 5: Effect of the Synergy Between HA Nanoparticles and Different Concentrations of Permeable CPAs on Related Gene Expression in Vitrified Bovine GV Oocytes

**Figure 7:**
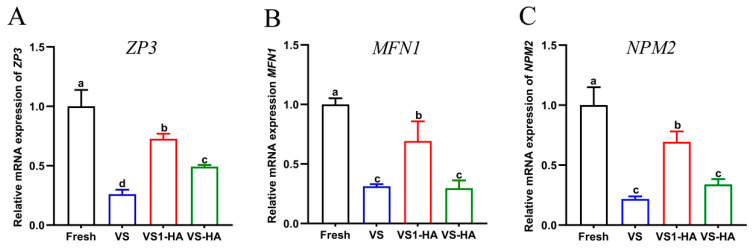
Expression levels of *ZP3* (**A**), *MFN1* (**B**), and *NPM2* (**C**) in vitrified–warmed bovine GV oocytes after IVM, analyzed by RT-qPCR across for four group: Fresh, VS, VS1-HA, and VS-HA. Data are presented as mean ± SD and normalized to *β-actin* using the 2^−∆∆Ct^ method. Analyses were performed in triplicate. Different lowercase letters (a, b, c, d) indicate statistically significant differences in gene expression (*p* < 0.05). Experimental groups: Fresh, non-vitrified control; VS, vitrified control without HA nanoparticles (CPAs: 20% EG + 20% DMSO); VS1-HA, vitrified with 17.5% EG, 17.5% DMSO, and 40 nm 0.05% HA nanoparticles; VS-HA, vitrified with 20% EG, 20% DMSO, and 40 nm 0.05% HA nanoparticles.

Gene expression results are presented in Figure 7. The relative expression levels of *ZP3*, *MFN1*, and *NPM2* were significantly higher in the VS1-HA group compared with the VS and VS-HA groups (*p* < 0.05). While *ZP3* expression was also higher in the VS-HA group than in the VS group (*p* < 0.05), no significant differences were observed between these two groups for *MFN1* and *NPM2* (*p* > 0.05). These results indicate that HA nanoparticles, in combination with low-concentration permeable CPAs, increased the expression of *ZP3*, *MFN1*, and *NPM2* in vitrified bovine GV oocytes after IVM, thereby providing protection oocytes against vitrification-induced damage.

## 4. Discussion

HA nanoparticles possess favorable biological properties, including high biosafety [22], moderate thermal conductivity, and excellent biocompatibility [19]. Previous studies have also shown that HA nanoparticles can increase the viscosity of vitrification solutions [20]. Consequently, incorporating HA nanoparticles of appropriate particle size and concentration into the vitrification solution, while simultaneously reducing the concentration of permeable CPAs, may represent a promising strategy to enhance vitrification effect in bovine GV oocytes.

Before application, it is essential to assess whether HA nanoparticles can cross biological barriers and demonstrate favorable biocompatibility [31]. In this study, TEM analysis revealed that HA nanoparticles were predominantly localized on and around lipid droplets and mitochondria, supporting their potential as a novel cryoprotectant for vitrified bovine GV oocytes. Nanoparticles typically enter the oocytes via endocytosis or membrane interactions, a common uptake pathway for nanomaterials in mammalian cells [32,33], which may explain their intracellular localization observed here. Appropriate nanoparticle dispersion is critical for effective oocyte uptake. Ultrasonic treatment improved HA nanoparticle dispersion, whereas prolonged ultrasonication reduced stability, likely due to overheating or re-agglomeration [34,35]. Particle size also affected dispersion behavior: smaller nanoparticles (20 nm) tended to aggregate rapidly due to dominant van der Waals forces, while larger nanoparticles (60 nm) experienced accelerated gravitational sedimentation because of greater mass, potentially reducing suspension duration and bioavailability [36,37,38]. Nanoparticle concentration is another key factor influencing biological effects. Previous studies indicate that optimal HA nanoparticle concentrations inhibit recrystallization during rewarming and stabilize vitrification solutions [28], whereas excessively high concentrations may impair oocyte development [22], and insufficient concentrations may limit bioavailability. Consistent with these findings, HA nanoparticles did not adversely affect GV oocytes during vitrification–warming [27], and at suitable particle sizes and concentrations, they enhanced mitochondrial activity and subsequent developmental competence. Similar benefits have been reported in other livestock species, where HA nanoparticles improved the development of vitrified oocytes [22,27]. Taken together, these results demonstrate that HA nanoparticles at appropriate particle sizes and concentrations exhibit favorable biocompatibility and cryoprotective potential, highlighting their promise as additives to improve the developmental competence of vitrified bovine GV oocytes.

The most commonly used permeable CPAs in vitrification protocols are EG and DMSO, typically applied at high concentrations (20% [*v*/*v*]) with LN as the cryogen [39]. However, such high concentrations of permeable CPAs can compromise oocytes due to osmotic stress during vitrification and warming, as well as intrinsic chemical toxicity [40,41]. To mitigate these adverse effects, HA nanoparticles were added to the vitrification solution while simultaneously reducing the concentration of permeable CPAs. These findings suggest that combining HA nanoparticles with lower concentrations of permeable CPAs exerts synergistic protective effects, thereby improving the developmental competence of vitrified bovine GV oocytes. This protective effect may be attributed to the relative viscosity [24] and thermal conductivity [25] of HA nanoparticles. High concentrations of permeable CPAs play a crucial role in the extracellular environment during vitrification and warming by preventing ice crystal formation [42,43]. The addition of HA nanoparticles reduces the dependence on high CPA concentrations, while their synergistic effect with lower concentrations of permeable CPAs not only inhibits ice crystal formation but also mitigates chemical toxicity and osmotic stress in oocytes. Consequently, this synergy enhances MMP level and developmental competence in vitrified bovine GV oocytes. HA nanoparticles improve the thermal conductivity of vitrification solutions, thereby increasing the critical cooling and warming rates [25]. Similarly, Wu et al. [44] reported that increasing the cooling rate using liquid helium (LHe, −269 °C) in combination with reduced CPA concentrations improved the developmental capacity of vitrified bovine GV oocytes. Collectively, these findings indicate that HA nanoparticles, as a novel cryopreservation additive, can reduce reliance on high CPA concentrations by increasing solution viscosity and thermal conductivity, ultimately enhancing MMP level and developmental competence in vitrified bovine GV oocytes.

Cryoinjury during vitrification disrupts critical subcellular structures in oocytes, particularly mitochondria and zona pellucida, which are essential for energy production, fertilization competence, and developmental potential [45,46,47,48,49]. In the present study, supplementation with HA nanoparticles markedly alleviated ultrastructural damage in vitrified bovine GV oocytes after IVM, especially when combined with reduced concentrations of permeable CPAs. These improvements were accompanied by enhanced mitochondrial activity and better-preserved zona pellucida structures, suggesting that HA nanoparticles may compensate for lower CPA concentrations while mitigating their toxicity, thereby maintaining mitochondrial integrity and supporting normal fertilization processes during vitrification. Previous studies have similarly highlighted that preserving mitochondrial function and zona pellucida integrity is critical for maintaining the developmental competence of bovine oocytes [50,51]. Consistent with these reports, the results from Experiment 3 showed that cleavage rates were higher in the HA-treated groups compared with the VS group. Lipid droplets, as key energy storage organelles, are closely linked to mitochondrial metabolism and cellular stress responses [44]. The relatively reduced lipid droplet accumulation observed in the HA-treated group under low CPA concentrations may reflect metabolic adaptation or redistribution of lipids during vitrification and warming. In summary, these findings indicate that the combination of HA nanoparticles with lower concentrations of permeable CPAs effectively mitigates ultrastructural damage in vitrified bovine oocytes, thereby improving oocyte MMP level and enhancing developmental competence.

At the molecular level, the expression of genes related to zona pellucida structure (*ZP3*), mitochondrial dynamics (*MFN1*), and chromatin organization (*NPM2*) was assessed. The zona pellucida is critical for sperm binding and preventing polyspermy [52], mitochondria are essential for ATP production during fertilization and early embryonic development [53,54], and proper maternal chromatin organization ensures correct pronuclear formation and embryonic competence [55,56]. In this study, supplementation with HA nanoparticles, particularly in combination with reduced concentrations of permeable CPAs (VS1-HA), was associated with significantly increased expression of *ZP3*, *MFN1* and *NPM2.* This suggests enhanced maintenance of zona pellucida biosynthesis mitochondrial transcriptional activity, and chromatin stability during vitrification. These molecular findings align with the observed improvements in mitochondrial function and ultrastructural integrity, indicating that the combined application of HA nanoparticles and lower CPA concentrations exerts synergistic protective effects on vitrified bovine GV oocytes, ultimately supporting improved developmental competence.

## 5. Conclusions

In summary, the synergistic combination of HA nanoparticles and lower concentrations of permeable CPAs effectively mitigated cryoinjury in vitrified bovine GV oocytes, preserving MMP level, developmental competence, ultrastructure, and the expression of *ZP3*, *MFN1*, and *NPM2*. These findings highlight HA nanoparticles as a valuable cryopreservation additive for enhancing vitrification efficiency of bovine oocytes. However, given the potential antioxidant properties of HA nanoparticles, future studies should investigate oxidative stress markers (e.g., MDA, ROS levels) and related gene expression to clarify the mechanisms underlying HA-mediated oocyte protection during vitrification and warming. Additionally, embryo transfer trials assessing implantation efficiency and in vivo developmental outcomes are warranted to validate the developmental competence of embryos derived from oocytes treated with this combined HA nanoparticle and low-CPA vitrification strategy.

## Figures and Tables

**Table 1:** Summary of experiment groups used in Experiments 1–5.

Group	Vitrification Solutions	Application
Fresh	None	Non-vitrified control, Experiment 1–5
VS	VS: 60% (*v*/*v*) HM, 20% (*v*/*v*) EG, 20% (*v*/*v*) DMSO, and 0.5 M sucrose	Vitrified control, Experiment 1–5
VH20	VS + 0.1% 20 nm HA nanoparticles	Experiment 1
VH40	VS + 0.1% 40 nm HA nanoparticles	Experiment 1
VH60	VS + 0.1% 60 nm HA nanoparticles	Experiment 1
VH-0.01%	VS + 0.01% 40 nm HA nanoparticles	Experiment 2
VH-0.05%	VS + 0.05% 40 nm HA nanoparticles	Experiment 2
VH-0.1%	VS + 0.1% 40 nm HA nanoparticles	Experiment 2
VS1	VS1: 70% (*v*/*v*) HM with 17.5% (*v*/*v*) EG, 17.5% (*v*/*v*) DMSO and 0.5 M sucrose	Experiment 3
VS1-HA	VS1 + 0.05% 40 nm HA nanoparticles	Experiment 3–5
VS-HA	VS + 0.05% 40 nm HA nanoparticles	Experiment 3–5

**Table 2:** Primers used for RT-qPCR.

Gene	Forward Primer (5′-3′)	Product Size	Gene ID
*β-actin*	F: TCGGTTGGATCGAGCATTCC R: ACTGGCCCCTTCTCCTTAGA	171	NM_173979.3
*ZP3*	F: TGTCGATGCTGTAGCAAGGG R: ACGCCACGGTCATTCATCTT	164	NM_173974.3
*MFN1*	F: TAGTAGACAGTCCGGGCACA R: AGGCTTGGAAAGTCGCTCAT	164	NM_001206508.1
*NPM2*	F: ACTGTGTGCTGTTGCTCAGT R: AACTCCAACCCCAGAACGAC	169	NM_001168706.1

**Table 3:** Effects of different concentrations HA nanoparticles on developmental competence of vitrified–warmed bovine GV oocytes.

Group	No. of Oocytes	Maturation Rate % (No.)	Cleavage Rate % (No.)	Blastocyst Rate % (No.)
Fresh	138	89.13 ± 2.64 ^a^ (123)	62.32 ± 3.29 ^a^ (86)	28.99 ± 2.48 ^a^ (40)
VS	131	39.69 ± 0.91 ^e^ (52)	12.21 ± 1.49 ^e^ (16)	3.05 ± 1.72 ^d^ (4)
VH-0.01%	138	42.03 ± 0.79 ^d^ (58)	15.22 ± 0.69 ^d^ (21)	5.07 ± 1.90 ^cd^ (7)
VH-0.05%	138	47.10 ± 1.03 ^b^ (65)	21.74 ± 1.19 ^b^ (30)	7.97 ± 1.04 ^b^ (11)
VH-0.1%	139	44.60 ± 1.10 ^c^ (62)	17.99 ± 1.22 ^c^ (25)	5.76 ± 1.17 ^c^ (8)

Values with different letters in the same column were significantly different (*p* < 0.05). Data are presented as mean ± SD. Experimental groups: Fresh, non-vitrified control; VS, vitrified control without HA nanoparticles; VH-0.01%, vitrified with 40 nm 0.01% HA nanoparticles; VH-0.05%, vitrified with 40 nm 0.05% HA nanoparticles; VH-0.1%, vitrified with 40 nm 0.1% HA nanoparticles.

**Table 4:** Effects of the synergy between HA nanoparticles and different concentrations of permeable CPAs on developmental competence of vitrified–warmed bovine GV oocytes.

Group	No. of Oocytes	Maturation Rate % (No.)	Cleavage Rate % (No.)	Blastocyst Rate % (No.)
Fresh	131	89.23 ± 2.72 ^a^ (117)	56.48 ± 3.46 ^a^ (74)	25.19 ± 2.70 ^a^ (33)
VS1	155	25.81 ± 1.89 ^e^ (40)	7.10 ± 2.77 ^e^ (11)	1.94 ± 1.64 ^e^ (3)
VS	128	38.55 ± 1.84 ^d^ (49)	10.94 ± 2.98 ^d^ (14)	3.13 ± 1.77 ^d^ (4)
VS1-HA	133	50.39 ± 2.12 ^b^ (67)	27.07 ± 2.88 ^b^ (36)	10.53 ± 1.62 ^b^ (14)
VS-HA	126	44.79 ± 1.09 ^c^ (56)	19.84 ± 1.75 ^c^ (25)	7.14 ± 1.64 ^c^ (9)

Values with different letters in the same column are significantly different (*p* < 0.05). Data are presented as mean ± SD. Experimental groups: Fresh, non-vitrified control; VS, vitrified control without HA nanoparticles (CPAs: 20% EG + 20% DMSO); VS1, vitrified with 17.5% EG and 17.5% DMSO, and without HA nanoparticles; VS1-HA: vitrified with 17.5% EG, 17.5% DMSO, and 40 nm 0.05% HA nanoparticles; VS-HA, vitrified with 20% EG, 20% DMSO, and 40 nm 0.05% HA nanoparticles.

## Data Availability

The original contributions presented in this study are included in the article. Further inquiries can be directed to the corresponding author.

## Abbreviations

The following abbreviations are used in this manuscript:

HA	Hydroxyapatite
GV	germinal vesicle
CPAs	Cryoprotectants
LN	Liquid nitrogen
MMP	Mitochondrial membrane potential
FBS	Fetal bovine serum
TEM	Transmission electron microscopy
HM	Holding medium
EG	Ethylene glycol
DMSO	Dimethyl sulfoxide
VS	Vitrification Solution
OPS	Open pulled straw
IVM	In Vitro Maturation
IVF	In Vitro Fertilization
IVC	In Vitro Culture
PVS	Perivitelline space
Mv	Microvilli
Mi	Mitochondria
ZP	Zona pellucida
LHe	Liquid helium
